# MDA-9/Syntenin regulates differentiation and angiogenesis programs in head and neck squamous cell carcinoma

**DOI:** 10.18632/oncoscience.99

**Published:** 2014-09-18

**Authors:** Regina A. Oyesanya, Shilpa Bhatia, Mitchell E. Menezes, Catherine I. Dumur, Karan P Singh, Sejong Bae, Dean A. Troyer, Robert B. Wells, Edward R. Sauter, David Sidransky, Paul B. Fisher, Oliver J. Semmes, Santanu Dasgupta

**Affiliations:** ^1^ Department of Human and Molecular Genetics, Virginia Commonwealth University, Virginia; ^2^ Department of Biology, Norfolk State University, Virginia; ^3^ Department of Pathology, Virginia Commonwealth University, Virginia; ^4^ University of Alabama at Birmingham Comprehensive Cancer Center's Biostatistics and Bioinformatics Shared Facility, University of Alabama at Birmingham, Alabama; ^5^ The Leroy T. Canoles Jr. Cancer Research Center, Eastern Virginia Medical School, Virginia; ^6^ Department of Pathology, University of Texas Health Science Center at Tyler, Texas; ^7^ Department of Surgery, University of Texas Health Science Center at Tyler, Texas; ^8^ Department of Otolaryngology and Head-Neck Surgery, The Johns Hopkins School of Medicine, Maryland; ^9^ Department of Cellular and Molecular Biology, University of Texas Health Science Center at Tyler, Texas

**Keywords:** Head and neck suqamous cell carcinoma, MDA-9/Syntenin, differentiation, SPRR1B, VEGFR1

## Abstract

Little is known about the molecular pathways regulating poor differentiation and invasion of head and neck squamous cell carcinoma (HNSCC). In the present study, we aimed to determine the role of MDA-9/Syntenin, a metastasis associated molecule in HNSCC tumorigenesis. Elevated MDA-9/Syntenin expression was evident in 67% (54/81) primary HNSCC tumors (p=0.001-0.002) and 69% (9/13) pre-neoplastic tissues (p=0.02-0.03). MDA-9/Syntenin overexpression was associated with the stage (p=0.001), grade (p=0.001) and lymph node metastasis (p=0.0001). Silencing of MDA-9/Syntenin in 3 poorly differentiated HNSCC cell lines induced squamous epithelial cell differentiation, disrupted angiogenesis and reduced tumor growth *in vitro* and *in vivo*. We confirmed SPRR1B and VEGFR1 as the key molecular targets of MDA-9/Syntenin on influencing HNSCC differentiation and angiogenesis respectively. MDA-9/Syntenin disrupted SPRR1B expression interacting through its PDZ1 domain and altered VEGFR1 expression *in vitro* and *in vivo*. VEGFR1 co-localized with MDA-9/Syntenin in HNSCC cell lines and primary tumor. Downregulation of growth regulatory molecules CyclinD1, CDK4, STAT3, PI3K and CTNNB1 was also evident in the MDA-9/Syntenin depleted cells, which was reversed following over-expression of MDA-9/Syntenin in immortalized oral epithelial cells. Our results suggest that early induction of MDA-9/Syntenin expression influences HNSCC progression and should be further evaluated for potential biomarker development.

## INTRODUCTION

Head and neck squamous cell carcinoma (HNSCC) is the sixth most common malignancy worldwide with an annual incidence of 600,000 cases [1]. In the United States, an estimated 10, 000 deaths result from 50,000 cases per year [1]. Intake of tobacco and related products, alcohol and high-risk human papilloma virus infection were identified as the major risk factors associated with HNSCC [1-2]. Recent studies have linked alterations of CyclinD1/CDKs, RB, TGF-beta, Tp53, EGFR, RAS, PIK3CA, p16, p63 and NOTCH1 signaling pathways with HNSCC proliferation, lack of differentiation, and survival [1]. However, identification of the molecular drivers behind HNSCC invasion and poor differentiation, and understanding their precise role remains largely unknown [1].

MDA-9/Syntenin is a PDZ domain-containing scaffold protein with an emerging role in tumorigenesis in the context of invasion and metastasis [3-4]. Overexpression of MDA-9/Syntenin has been reported in multiple human cancer cell lines, advanced primary tumors associated with increased cell migration/invasion and as a predictor of metastasis of colorectal cancer [3-5]. Cross-talk between MDA-9/Syntenin, c-Src, NF-kB and EGFR favoring invasion and metastasis was demonstrated in melanoma and urothelial carcinomas [3-4]. However, the precise role of MDA-9/Syntenin and its molecular partners in HNSCC progression are largely unknown.

We observed overexpression of MDA-9/Syntenin in pre-neoplastic, primary and metastatic tumors from human HNSCC patients and a positive correlation with HNSCC progression. Silencing of MDA-9/Syntenin expression in 3 established HNSCC cell lines induced squamous epithelial cell differentiation, disrupted angiogenesis and reduced tumor growth *in vitro* and *in vivo*. We confirmed SPRR1B and VEGFR1 as the key molecular partners of MDA-9/Syntenin on influencing HNSCC differentiation and angiogenesis respectively. Downregulation of the growth regulatory molecules CyclinD1, CDK4, STAT3, PI3K and CTNNB1 was also evident in these cells following the silencing of MDA-9/Syntenin. Forced over-expression of MDA-9/Syntenin in immortalized oral epithelial cells resulted in increased cellular proliferation accompanied with enhanced expression of the above growth regulatory molecules, VEGFR1 and decreased expression of SPRR1B. We also identified a number of additional differentiation, angiogenic and receptor tyrosine kinase molecules, which were downmodulated following the silencing of MDA-9/Syntenin in the HNSCC cells. This study reveals a novel role for MDA-9/Syntenin on influencing HNSCC differentiation and angiogenesis in concert with SPRR1B and VEGFR1. MDA-9/Syntenin could be an attractive target to develop early detection and monitoring strategies for HNSCC.

## RESULTS

### Elevated expression of MDA-9/Syntenin in premalignant to malignant HNSCC

**Figure 1:**
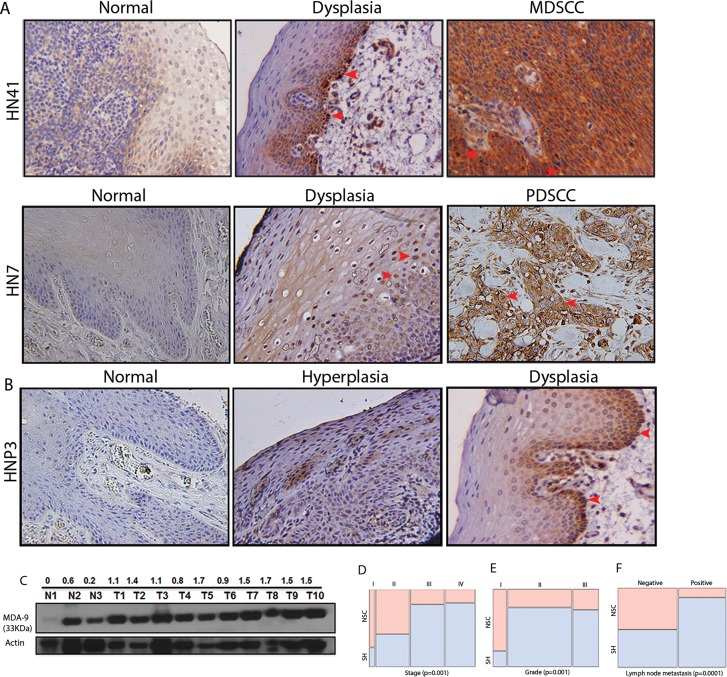
MDA-9/Syntenin expression pattern in HNSCC progression (A) Progressive increase in MDA-9/Syntenin expression was evident from normal to pre-neoplastic to corresponding metastatic squamous cell carcinoma (p=0.001-0.002). Both cytoplasmic and nuclear expression (red arrow heads) of MDA-9/Syntenin was evident in the dysplasia and the primary tumors. Magnification X 200. (B) MDA-9/Syntenin expression was also higher (p=0.02-0.03) in hyperplasia and dysplasia compared to normal squamous epithelial tissues. Magnification X 200. (C) Elevated expression level of MDA-9/Syntenin in primary HNSCC tumors by Western-blotting. Actin was used as loading control. (D-F) MDA-9/Syntenin expression was significantly associated with stage (p=0.001, D), grade (p=0.001, E) and lymph node metastasis (p=0.0001, F). Grade-I: WDSCC; grade-II: MDSCC; grade-III: PDSCC.

HNSCC is a frequently recurring disease with loco-regional relapse and poor differentiation pattern [6]. We recently demonstrated MDA-9/Syntenin as a key component of invasion and metastasis in uroepithelial cell carcinomas [4]. To investigate, whether MDA-9/Syntenin plays any role in the growth and invasion of oral epithelial cell carcinomas, as the first step, we measured MDA-9/Syntenin protein expression in 81 primary HNSCC tumors from various stages including tumors positive for lymph node metastasis (Table S1). All cases were SCC and had corresponding adjacent normal tissues. Immunohistochemistry (IHC) analysis confirmed higher (p=0.001-0.002) expression of MDA-9/Syntenin in 67% (54/81) of the primary tumors (Table S1 and Figure 1A). Two cases (HN41 and HN7, Table S1) had corresponding pre-neoplastic tissue along with their primary tumors where a progressive increase of MDA-9/Syntenin was evident (Figure 1A). We also detected an enhanced level of MDA-9/Syntenin expression (p=0.02-0.03) in 69% (9/13) of the pre-neoplastic squamous epithelium compare to their normal counterpart (Figure 1B). Both nuclear (arrowheads) and cytoplasmic localization of MDA-9/Syntenin was evident in the dysplastic epithelium and the carcinomas (Figure 1A-B). Western blot analysis confirmed elevated expression level of MDA-9/Syntenin (1-4 folds) in 10 primary HNSCC tumors positive for lymph node metastasis, when compared to 3 normal (unpaired) tissues (Figure 1C). MDA-9/Syntenin expression was associated with stage (p=0.001), grade (p=0.001) and lymph node metastasis (p=0.0001) (Figure 1D-F). Thus, MDA-9/Syntenin appears to be elevated and associated with HNSCC progression.

### MDA-9/Syntenin depletion halted HNSCC growth and invasion *in vitro* and *in vivo*

**Figure 2:**
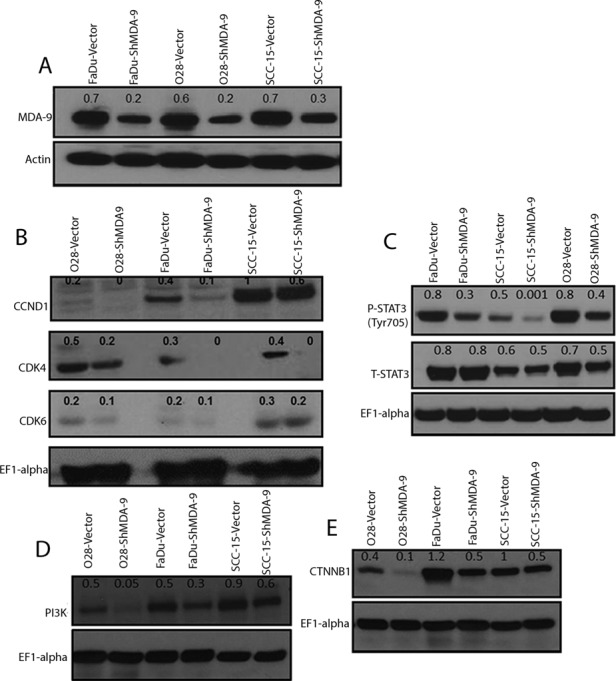
Impact of silencing of MDA-9/Syntenin in HNSCC cells (A) Effective knockdown of MDA-9/Syntenin was evident in 3 established HNSCC cell lines. Actin was used as a loading control. Marked downregulation of cell cycle regulatory molecules CCND1 and CDK4 (B), activated STAT3 (Tyr705, C) was evident by Western-blot analysis in the MDA-9/Syntenin depleted HNSCC cells as indicated. Decreased expression of PI3K (D) and CTNNB1 (E) was also evident by Western-blot analysis in the MDA-9/Syntenin depleted HNSCC cells.

**Figure 3:**
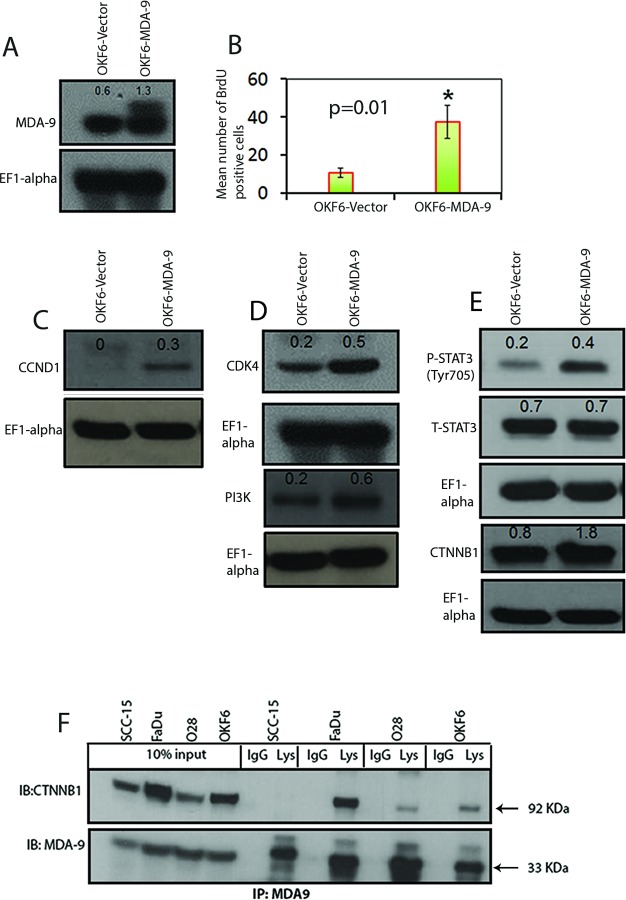
Effect of MDA-9/Syntenin overexpression on immortalized oral keratinocytes (A) MDA-9/Syntenin overexpression in the OKF6 cells as indicated. (B) Overexpression of MDA-9/Syntenin in OKF6 cells resulted in a significant increase in proliferation (p=0.01), compared to the empty vector treated group. (C-E) Marked upregulation of CCND1 (C), CDK4, PI3K (D), activated STAT3 (Tyr705) and CTNNB1 (E) in the MDA-9/Syntenin-transfected cells compared to the empty vector-treated cells as indicated. EF1-alpha was used as a loading control. (F) Co-immunoprecipitation of MDA-9/Syntenin and CTNNB1 in all but one oral epithelial cell lines. IgG: Control immunoglobulin G, Lys: Whole cell lysate used for IP analysis. IB: Immunoblotting; IP: Immunoprecipitation.

The magnitude of MDA-9/Syntenin expression and its association with HNSCC progression suggest a functional role for MDA-9/Syntenin in HNSCC tumorigenesis. To determine the impact of MDA-9/Syntenin abrogation on HNSCC growth and progression, we stably depleted MDA-9/Syntenin expression in 3 aggressive and poorly differentiated HNSCC cell lines FaDu, SCC-15 and O28 (Figure 2A). Since we observed similar level of depletion of MDA-9/Syntenin in different clones, a single clone was selected from each HNSCC cell line for subsequent analyses [4]. We observed decreased *in vitro* proliferation (Figure S1A, p=0.004-0.02), invasion (Figure S1B, p=0.01-0.02) and anchorage independent growth (Figure S2, p=0.02-0.04) in all the MDA-9/Syntenin depleted groups compared to the empty vector treated groups. Western blot analysis of MDA-9/Syntenin depleted HNSCC cells demonstrated marked reduction in the expression level of CyclinD1 (CCND1), CDK4, activated STAT3 (Tyr705), PI3K and CTNNB1 and slight decrease in CDK6 expression (Fig. 2B-E). Reversal of cellular proliferation (p=0.01) and accompanying increase in the expression level of CCND1, CDK4, activated STAT3 (Tyr705), PI3K and CTNNB1 were evident in the immortalized OKF6 cells following overexpression of MDA-9/Syntenin (Figure 3A-E). Co-immunoprecipitation of CTNNB1 and MDA-9/Syntenin was reported earlier [7]. We could pull down CTNNB1 by MDA-9/Syntenin in all but all the oral epithelial cell lines (Figure 3F).

**Figure 4:**
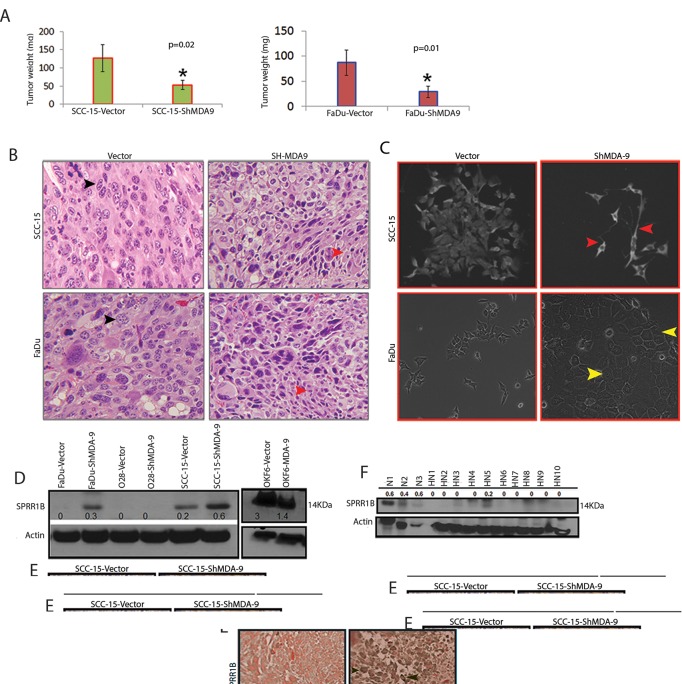
Effect of MDA-9/Syntenin depletion on tumor growth and differentiation of HNSCC cells (A) MDA-9/Syntenin depleted HNSCC xenografts exhibited decreased tumor growth (p=0.01-0.02). (B) Empty vector treated cells appeared poorly differentiated with more nuclear pleomorphism, larger nucleoli, and more clumped chromatin *in vivo* (left panel, black arrowheads) compared to the MDA-9/Syntenin depleted cells, which appeared moderately differentiated (right panel, red arrowheads). (C) Under *in vitro* condition, MDA-9/Syntenin depleted SCC-15 and FaDu cells also appeared more differentiated compared to the empty vector treated cells (right panel, red-and yellow arrowheads). Magnification X 200. (D-E) Restored expression of SPRR1B *in vitro* in all but one MDA-9/Syntenin depleted HNSCC cell lines (D) and SCC-15 xenograft (E). (F) SPRR1B expression was low or barely detectable by Western blotting in primary HNSCC tumor positive for lymph node metastasis (Table S1). Actin was used as loading control.

To determine the impact of MDA-9/Syntenin silencing on HNSCC growth *in vivo,* we implanted MDA-9/Syntenin depleted FaDu and SCC-15 cells subcutaneously in athymic nude mice. Under this *in vivo* setting, growth of the MDA-9/Syntenin depleted FaDu and SCC-15 xenografts was significantly reduced (p=0.01-0.02) compared to the empty vector treated groups (Figure 4A). A significant decrease in DNA-synthesis was also observed in the MDA-9/Syntenin depleted FaDu (Figure S3A, p=0.009) and SCC-15 (Figure S3B, p=0.014) xenografts compared to the empty vector treated groups. Similar to our *in vitro* observation, we confirmed a significant decrease in CCND1 expression level in MDA-9/Syntenin depleted FaDu and SCC-15 xenografts (Figure S4A, p=0.01-0.03). We also confirmed low expression of MDA-9/Syntenin in SCC-15 xenografts depleted of MDA-9/Syntenin (Figure S4B, p=0.01).

### MDA-9/Syntenin depletion induced differentiation of the HNSCC cells

MDA-9/Syntenin was initially discovered as a molecule associated with terminal differentiation (8). To understand the impact of MDA-9/Syntenin on HNSCC differentiation, we performed histopathological analysis (per pathologic guidance, RBW) of the MDA-9/Syntenin depleted FaDu and SCC-15 xenografts. We observed marked alteration in the differentiation pattern of the MDA-9/Syntenin depleted FaDu and SCC-15 cells compared to the controls (Figure 4B). Empty vector treated groups were poorly differentiated with more nuclear pleomorphism, larger nucleoli, and more clumped chromatin (Figure 4B, left panel black arrow heads) compared to the MDA-9/Syntenin depleted cells, which appeared moderately differentiated (Figure 4B, left panel and red arrow heads). The MDA-9/Syntenin depleted FaDu and SCC-15 cells also appeared more differentiated *in vitro,* compared to the empty vector treated groups (Figure 4C, arrow heads). A cDNA microarray analysis of MDA-9/Syntenin depleted FaDu cells (Gene Expression Omnibus; #GSE57760) demonstrated a 52.1 upregulation of the mRNA level of SPRR1B, a key molecule associated with squamous epithelial cell differentiation [9-12]. Restored SPRR1B protein expression was confirmed *in vitro* in all but one HNSCC cell lines depleted of MDA-9/Syntenin by Western-blotting (Figure 4D) and SCC-15 xenograft (Figure 4E, p=0.001). Reversal of SPRR1B expression was evident following overexpression of MDA-9/Syntenin in immortalized OKF6 cells (Figure 4D). Western blot analysis demonstrated a low or barely detectable level of SPRR1B expression in all the 10 primary HNSCC tumors (1 WDSCC, 6 MDSCC and 3 PDSCC, Table S1) exhibiting high level of MDA-9/Syntenin expression compared to the normal counterparts (Figure 4F). Notably, one of the normal tissues (N1) with the highest level of SPRR1B expression had a barely detectable level of MDA-9/Syntenin expression (Figure 1C and 4F).

### MDA-9/Syntenin regulates SPRR1B through the PDZ domain

**Figure 5:**
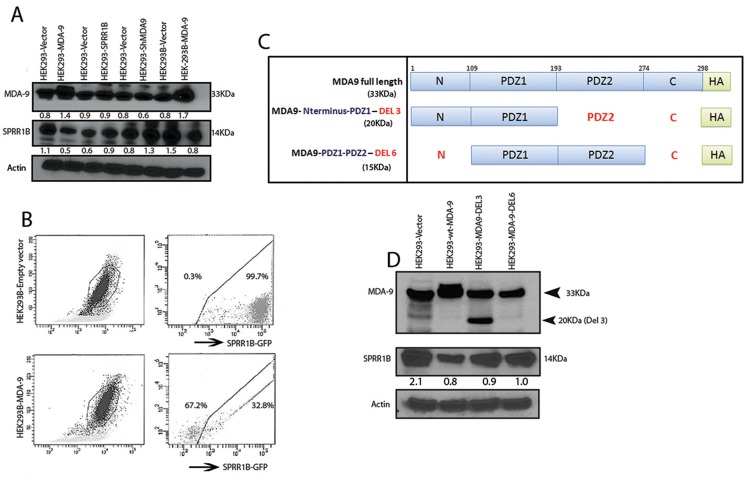
MDA-9/Syntenin regulation of SPRR1B (A) Stable overexpression of MDA-9/Syntenin in HEK293 cells (HEK293-MDA-9) markedly decreased SPRR1B expression compared to control (HEK293-Vector), but stable overexpression of SPRR1B in H3K293 cells (HEK293-SPRR1B) did not alter MDA-9/Syntenin expression compared to control (HEK293-Vector). SPRR1B expression was elevated following stable depletion of MDA-9 from HEK293 cells (HEK293-Sh-MDA-9) compared to control (HEK293-Vector). Overexpression of MDA-9/Syntenin in HEK293 cells stably overexpressing SPRR1B (HEK293B-MDA-9) reduced SPRR1B expression compared to the corresponding empty vector control (HEK293-Vector). Actin was used as loading control. (B) FACS analysis of the GFP-tagged SPRRB1 overexpressing HEK293 cells (denoted as HEK293B) revealed marked loss of GFP positive cells following overexpression of MDA-9/Syntenin compared to the empty vector transfected cells (32.8% vs. 99.7%). (C) The map of the various deletion constructs and the wild type MDA-9/Syntenin used to transfect HEK293 cells. (D) Overexpression of the wild type MDA-9/Syntenin or domains containing N-terminal plus PDZ1 or PDZ1 plus PDZ2 reduced SPRR1B expression in the HK293 cells as indicated. Actin was used as loading control.

MDA-9/Syntenin appears to influence the expression of SPRR1B (Fig. 4). To determine whether MDA-9/Syntenin directly influences SPRR1B expression or vice versa, we stably overexpressed or depleted MDA-9/Syntenin in HEK293 cells or HEK293 cells stably overexpressing SPRR1B. Overexpression of MDA-9/Syntenin in HEK293 cells resulted in marked reduction of SPRR1B expression (Figure 5A). Stable overexpression of SPRR1B in HEK293 cells did not reduce MDA-9/Syntenin expression (Figure 5A). Depletion of MDA-9/Syntenin from the HEK293 cells resulted in marked increase of SPRR1B expression (Figure 5A). On the other hand, overexpression of MDA-9/Syntenin on HEK293 cells stably overexpressing SPRR1B (HEK293B) markedly reduced SPRR1B expression (Figure 5A). We also examined the impact of MDA-9/Syntenin overexpression on SPRR1B expression by FACS analysis as the exogenous SPRR1B expression was traceable through the attached GFP-tag in the HEK293 cells (Figure 5B). Frequency of the GFP positive cells reduced markedly following the overexpression of MDA-9/Syntenin in the HEK293 cells overexpressing SPRR1B (HEK293B, 32.8% *vs.* 99.7%, Figure 5B).

MDA-9/Syntenin is known to interact with various molecules through the PDZ1 and PDZ2 domains [3-4, 7]. To identify the possible interacting domain(s) of MDA-9/Syntenin for SPRR1B regulation, we overexpressed two deletion mutants of MDA-9/Syntenin expressing the N-terminal plus PDZ1 or the PDZ1 plus PDZ2 domains along with the *wild type* version in HEK293 cells (Figure 5C). We analyzed the pooled clones for SPRR1B expression after antibiotic selection for two week. Along with the wild type (33KDa), overexpression of N-terminal plus PDZ1 domain of MDA-9/Syntenin (20KDa) resulted in marked reduction of SPRR1B expression (Figure 5D, lane 1-3). We also observed a similar pattern of decrease in the expression of SPRR1B following overexpression of MDA-9/Syntenin deletion mutant containing the PDZ1 plus PDZ2 domain (Figure 5D, lane 4). These results suggest PDZ1 as the potential domain responsible for SPRR1B regulation. Other than SPRR1B, cDNA microarray analysis of the MDA-9/Syntenin depleted FaDu cells (GEO GSE #57760) identified a 72-fold upregulation of KRT6A, another molecule involve in the regulation of HNSCC differentiation [11]. We confirmed the restored expression level of KRT6 (p=0.0008) by immunohistochemistry in FaDu and SCC-15 xenografts depleted of MDA-9/Syntenin (Figure S5).

### Silencing of MDA-9/Syntenin abrogated angiogenesis through the disruption of VEGFR1

**Figure 6:**
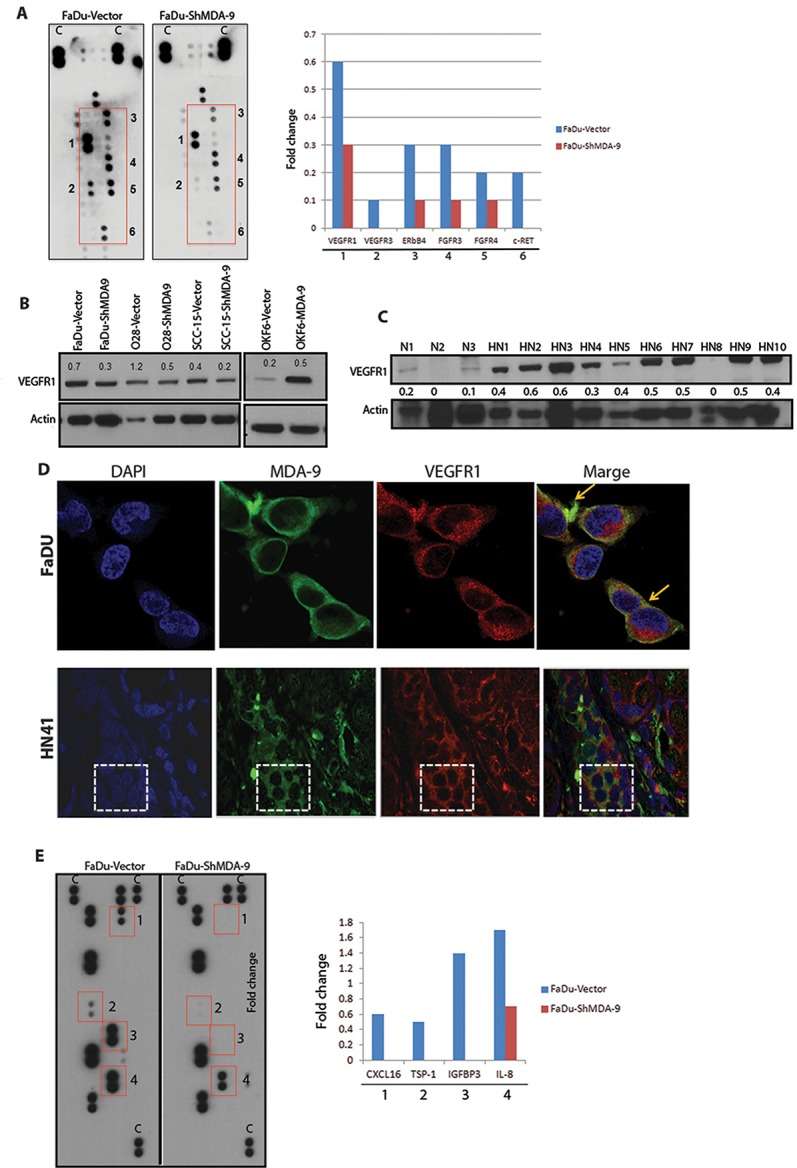
Impact of MDA-9 depletion on angiogenesis of HNSCC (A) A receptor tyrosine kinase array revealed a marked decrease in the level of expression of VEGFR1, VEGFR3, ERbB4, FGFR3 and c-RET in the MDA-9/Syntenin depleted FaDu cells (left panel) and indicated as fold change in the bar diagram (right panel). (B) Decreased or increased VEGFR1 expression following depletion or overexpression of MDA-9/Syntenin in HNSCC and immortalized oral epithelial cells respectively. (C) High VEGFR1 expression in all but one (HN8) primary HNSCC tumors positive for lymph node metastasis (Table S1). (D) Co-localization of MDA-9/Syntenin and VEGFR1 (arrows) was evident in FaDu cells and primary human HNSCC tumor (HN41). (E) Barely detectable expression level of CXCL16 (box 1), TSP-1 (box 2), IGFBP3 (box 3) and marked reduction in IL-8 (box 4) expression was observed in FaDu cells depleted for MDA-9/Syntenin compared to the control vector treated cells (left panel). C: control. The respective fold changes was shown in the bar diagram (right panel).

Various angiogenic factors and receptor tyrosine kinase (RTK) molecules including VEGFR1, VEGFR3 play a key role in HNSCC progression [1-2, 13-14]. To understand the impact of MDA-9/Syntenin silencing on the expression of various RTKs and angiogenic factors, we performed an RTK and angiogenesis array on MDA-9/Syntenin depleted FaDu cells. The RTK array analysis identified markedly reduced expression of VEGFR1, VEGFR3, ERBb4, FGFR4 and c-RET in the MDA-9/Syntenin depleted FaDu cells (Figure 6A). Since VEGFR1 plays a pronounced role in HNSCC progression [13-14], we further evaluated VEGFR1 expression level in the MDA-9/Syntenin depleted and overexpressing cells. Decreased level of VEGFR1 was confirmed in all the MDA-9/Syntenin depleted HNSCC cell lines *in vitro* (Figure 6B), which was reversed following overexpression of MDA-9/Syntenin in immortalized OKF6 cells (Figure 6B). A high level of VEGFR1 expression was evident in 10 primary HNSCC tumors exhibiting elevated level of MDA-9/Syntenin (Fig. 6C). Significantly decreased VEGFR1 expression (p=0.001-0.003) was confirmed in MDA-9/Syntenin depleted FaDu and SCC-15 xenografts (Figure S6A). We also observed co-localization of MDA-9/Syntenin and VEGFR1 in FaDu cells and one primary HNSCC tumor HN41 positive for lymph node metastasis (Figure 6D, Table S1).

The angiogenesis array analysis revealed barely detectable level of CXCL16, TSP1, IGFBP3 and markedly decreased level of IL-8 in the MDA-9/Syntenin depleted FaDu cells (Figure 6E). On the other hand, the HUVAC cell assay exhibited marked disruption in the capillary tube formation *in vitro* when treated with conditioned medium obtained from MDA-9/Syntenin depleted HNSCC cell lines (Figure S6B). The mean vessel density as determined by the CD31 positivity was significantly decreased into the tumor surrounding stroma (Figure S7A, p=0.004-0.01) and the tumor beds (Figure S7B, p=0.001-0.009) of the MDA-9/Syntenin depleted FaDu and SCC-15 xenografts compared to their respective empty vector treated controls.

## DISCUSSION

Head and neck squamous cell carcinoma is a frequently recurrent disease with high mortality and limited treatment options beyond the conventional surgery and chemo/radiation therapy [1]. Little is known about the factors driving HNSCC invasion and poor differentiation despite the recent advancement [1]. The present study identified MDA-9/Syntenin as a molecule, overexpressed in primary and metastatic HNSCC tumors and influence HNSCC differentiation and angiogenesis.

MDA-9/Syntenin is located on human chromosome 8q (8q12), a frequently amplified region in HNSCC [15-16]. To understand the mechanism behind overexpression of MDA-9/Syntenin in the primary HNSCC tumors, we performed FISH analysis [4]. However, similar to our observation in uroepithelial cell carcinomas [4], we could not detect any amplification in 40 paired HNSCC cases analyzed (data not shown). Thus, other potential mechanism(s) such as activating mutation, miRNA regulation or regulation through other interacting molecules could be involved behind the elevated expression of MDA-9/Syntenin in the progressive stages of HNSCC. Somatic mutations within MDA-9/Syntenin in carcinomas of the colon, kidney, uterine cervix and breast are evident in the catalogue of somatic mutation in cancer database (COSMIC, http://cancer.sanger.ac.uk/). We have detected one somatic mutation within the coding regions of MDA-9/Syntenin in one HNSCC patient (HN41), which could be an activating mutation and possibly explains the reason for the progressive elevation of MDA-9/Syntenin from premalignant to the corresponding malignant lesion observed in this patient (unpublished observation). On the other hand, elevated MDA-9/Syntenin expression in premalignant lesions and its favorable impact on the growth of the immortalized oral epithelial cells following its overexpression, strongly suggest that early upregulation is beneficial to malignant transformation and progression.

Silencing of MDA-9/Syntenin reduced tumor burden in mice. Mechanistically, we observed a repression of key HNSCC growth associated molecules including CCND1/CDK4 [1], as well as upregulation of the differentiation factor SPRR1B following knockdown of MDA-9/Syntenin. SPRR1B (a.k.a cornifin) belongs to small-proline-rich protein family and is a squamous cell differentiation marker [9-12]. In GEO database, marked reduction of SPRR1B mRNA expression was evident in 68% (15/22) of primary HNSCC tumors compared to their corresponding normal (GEO #GDS2550), which supports our finding of barely detectable level of SPRR1B protein in moderate to poorly differentiated primary tumors and cell lines. Loss of SPRR1B was associated with the development of bronchial epithelial cells malignancy and skin squamous cells carcinomas [9-10]. Downregulation of SPRR1B was reported in poorly differentiated scirrhous gastric epithelial cancer cells with high metastatic potential [12]. Moreover, overexpression of SPRR1B was shown to augment entry of cells into the G0 phase of the cell cycle [17]. Possibly as a result, we have observed decreased DNA synthesis and proliferative activities of the MDA-9/Syntenin depleted HNSCC cells with restored SPRR1B expression. Recent studies by us and others have demonstrated the emerging role of MDA-9/Syntenin in regulating cellular proliferation in uroepithelial and breast cancer through various mechanisms [4, 18]. We could not detect SPRR1B expression in the most aggressive and poorly differentiated HNSCC cell line O28, indicating its unrecoverable loss favoring progression.

Perturbation of SPRR1B expression by the wt-MDA-9/Syntenin, through the PDZ1 containing domain, suggests that MDA-9/Syntenin mediates HNSCC terminal differentiation via SPRR1B expression. Our observation that MDA-9/Syntenin overexpression was evident in pre-neoplastic stages supports a model in which MDA-9/Syntenin mediated down-regulation of SPRR1B is an early event preventing terminal differentiation thereby aiding in tumor progression. Failure in normal differentiation was proposed as an important step in malignant transformation [10] and thus loss of normal differentiation regulators may allow tumor development and progression.

Other than SPRR1B, we cannot also rule out the possibility of the involvement of other squamous cell differentiation factors KRT6A, SPRR1A, KRT14 and KRT5 on inducing HNSCC differentiation [10]. These factors had 71.4, 40.4, 17.9 and 13.7 fold upregulation of their mRNA expression respectively (GEO GSE #57760) following the depletion of MDA-9/Syntenin in the HNSCC cells. Restoration of KRT6A expression in MDA-9/Syntenin depleted HNSCC xenografts further support the above notion. Thus, MDA-9/Syntenin appears to play an important role in inhibiting squamous epithelial cell differentiation thereby favoring HNSCC progression. An earlier study demonstrated CTNNB1 induced loss of differentiation of differentiated mammary epithelial cells pointing towards it role in epithelial differentiation regulation and determining cells fate [19]. Beta-catenin was also found to co-localize with MDA-9/Syntenin [7]. Thus, co-immunoprecipitation of CTNNB1 and MDA-9/Syntenin, and alteration of CTNNB1 following MDA-9/Syntenin depletion or overexpression suggest for a MDA-9/Syntenin and CTNNB1 signaling axis in regulating HNSCC differentiation in concert with SPRR1B. Notably, MDA-9/Syntenin expression was detectable in the nucleus other than the adherens junction in the primary HNSCC tumors. However, it remains to be determined whether an interaction between MDA-9/Syntenin and CTNNB1 has occurred in the nucleus.

Various RTKs and non-RTK family of angiogenic and growth regulatory factors including VEGFR1, ERbB4, FGFR3/4, c-RET, CXCL16, TSP-1, IGFBP3 and IL-8 are involved and implicated in the overwhelming network of HNSCC angiogenesis, proliferation, invasion/metastasis and therapeutic resistance [20-30]. VEGFR1 in particular is frequently overexpressed and associated with increased angiogenesis, proliferation and poor survival of HNSCC patients [14]. Co-localization of VEGFR1 with MDA-9/Syntenin, high co-expression in primary tumors and its ablation following MDA-9/Syntenin silencing strongly suggest for their functional coordination to regulate angiogenesis in HNSCC. Possibly due to the disruption of VEGFR1 signaling in concert with the downregulation of other angiogenic and growth regulatory molecules ERbB4, FGFR3/4, c-RET, CXCL16, TSP-1, IGFBP3 and IL-8, we observed disrupted intratumoral and tumor surrounding stromal vasculature, capillary tube formation and hence halted HNSCC progression. Regional lymph node metastasis and lymphangiogenesis are the key components of HNSCC progression and significant predictors for the aggressiveness and outcome [31]. VEGFR3 was identified as a key factor associated with HNSCC lymphangiogenesis [31-32]. A positive correlation between MDA-9/Syntenin expression and lymph node metastasis and disruption of VEGFR3 in MDA-9/Syntenin depleted HNSCC cells is suggestive of a functional link between MDA-9/Syntenin and VEGFR3.

**Figure 7:**
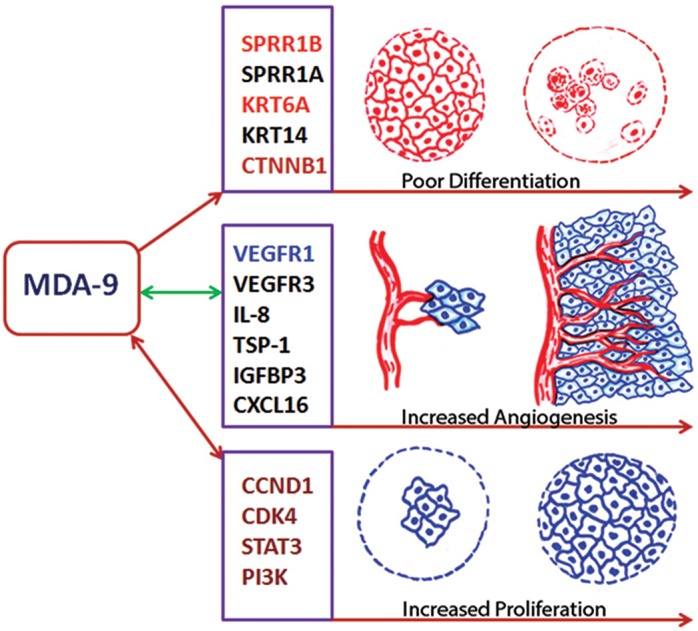
MDA-9/Syntenin regulation of HNSCC progression MDA-9/Syntenin alters differentiation, angiogenesis and proliferation of HNSCC via interaction with various molecules as indicated. Molecules exhibited in colors were confirmed for their involvement in HNSCC progression in concert with MDA-9/Syntenin. One sided arrow suggest for an MDA-9/Syntenin mediated disruption of the squamous differentiation factor(s), where as both sided arrows suggests for a cross talk between MDA-9/Syntenin and various angiogenesis and proliferation associated molecules.

Taken together, we uncovered a novel role of MDA-9/Syntenin in HNSCC differentiation and angiogenesis. The resulting impact on HNSCC tumor development is mediated through the regulation of various squamous differentiation, proliferation and angiogenic factors including SPRR1B and VEGFR1 (Fig. 7). Many other molecules associated with HNSCC differentiation and angiogenesis were also altered following MDA-9/Syntenin depletion (Fig. 7) and our laboratory is currently examining their potential role. Based on our findings, MDA-9/Syntenin should be further explored for its potential to develop novel HNSCC detection and surveillance strategies.

## MATERIALS AND METHODS

### Tissue samples and ethical statement

Pre-constructed tissue microarray slides containing primary tumors and corresponding adjacent normal tissues or fresh frozen paired normal/tumor HNSCC tissues were purchased from commercial sources (US Biomax Inc.) and from the Virginia Commonwealth University (VCU) Tissue and Data Acquisition and Analysis Core (TDAAC) facility per IRB approved protocol. A panel of pre-neoplastic oral squamous tissues microarray (de-identified) containing hyperplasitc and dysplastic lesion was procured from the laboratory of Dr. Edward Sauter, Department of Surgery, The University of Texas Health Science center at Tyler. The demographic data of all the HNSCC patients along with MDA-9/Syntenin expression patterns are represented in Table S1.

### Cell lines and reagent

Authenticated FaDu, SCC-15 and HEK293 cells were purchased from ATCC and cultured in ATCC-recommended medium. Authenticated O28 and OKF6 cell lines were kind gift from the laboratory of Dr. David Sidransky, Johns Hopkins University. All cell lines were authenticated at the VCU core facility and periodically checked for mycoplasma contamination using a mycoplasma detection kit (Sigma # MP-0025). All tissue culture media and reagents were purchased either from ATCC or Invitrogen.

### Immunohistochemistry

Immunohistochemistry (IHC) was performed using specific antibody in paired primary HNSCC and pre-neoplastic tissue specimens as described above [4]. MDA-9/Syntenin antibody was obtained from Abnova Corporation (dilution 1:200). The EGFR, CCND1, CDK4/6, CTNNB1, PI3K, STAT3, EF1-alpha and Beta-actin antibodies were procured from Cell Signaling (dilution 1:200). The VEGFR1 and SPRR1B antibodies were procured from Abcam. Anti-mouse and rabbit secondary antibodies were obtained from Jackson Immunoresearch (dilution 1:1000). All IHC and histological evaluation was done per pathologist's guidance.

### Plasmid Constructions and transfections

The wild type (*wt)* MDA-9/Syntenin cDNA (Gene ID: 6386) was subcloned into SalI and NotI sites of the phosphorylated cytomegalovirus pCMV/myc/cyto plasmid, which has an N-terminal myc tag (Invitrogen) as described [4]. For knock down (KD) experiments, MDA-9/Syntenin-specific shRNA was constructed and cloned into the BamI-HindIII sites of pRNA3.1 vector (Genescript Corporation, Piscataway, NJ) as described earlier. Various MDA-9/Syntenin deletion constructs were also described previously [4].

### SPRRB1 transfection study

HEK293 cells were stably transfected with a GPF tagged SPRR1B plasmid (pCMV6-AC-GFP, # RG220108, Origene Inc.). In select experiment, SPRR1B overexpressing HEK293 cells were also transiently or stably transfected with wt-MDA-9 or ShMDA-9 plasmids as described above.

### Cell Proliferation Assay

Proliferation of the transfected cells plated in triplicate wells was determined by a BrdU incorporation assay kit (Roche Diagnostics) as per the manufacturer's protocol [4]. Data are presented as mean ± S.E. of duplicate experiments.

### Soft agar assay and Cell Invasion Assay

The anchorage independent growth capabilities of the transfected cells were determined by soft agar colony formation assay as described earlier [33]. Cell invasion capacity was assessed using the Cell Invasion Assay Kit (BD Biosciences) [4].

### Dual color fluorescence in situ hybridization (FISH)

Dual color fluorescence *in situ* hybridization (FISH) was performed on formalin-fixed paraffin-embedded (FFPE) sections obtained from 40 HNSCC patients (HN21-HN60, Table S1) as described earlier [4]. All tumors had corresponding normal tissue. Specimens were considered amplified for *mda-9/Syntenin* when they demonstrated nuclei containing numerous red test probe signals with a test probe: control (CEP) probe ratio >2 [4]. Cases showing an increased number of test to control probe signals, but in a ratio of >1.2 but < or equal to 2 were scored as a low level gain for that respective test probe. Cases, in which both the test and control probes were equally increased in number, were considered to show polysomy for chromosome 8 [4].

### Dual color Immunofluorescence and confocal microscopy

Cultured cells or FFPE tumor sections were fixed in 4% paraformaldehyde as described [4] and stained with both the MDA-9/Syntenin (Abnova, 1:200 dilution) and VERGF1 antibodies overnight at 4 0C followed by staining with FITC or Texas red-tagged secondary antibody (Jackson Immunoresearch, 1:1000 detail for all abs) for 1 hour at room temperature. Cells were then washed thoroughly with PBS and mounted with Prolong Gold antifade reagent (Invitrogen) and observed immediately under a confocal microscope. At least 10-fields were randomly selected for examining the staining intensity and distribution pattern of the proteins.

### *In vivo* DNA synthesis analysis

We performed IF analysis of the FFPE xenogratfs with anti-DNA antibody (Acris antibodies # BM5520P) to measure the level of DNA synthesis as described above. DAPI was used for counterstaining of the nucleus. At least 10-fields were randomly selected for counting the positively stained cells from multiple mice and data were represented as mean ± S.E.

### Western blotting (WB) and Co-Immunoprecipitation (Co-IP) analysis

Preparation of whole cell lysates and Western blotting analysis was performed following standard protocols as described earlier [4]. The Co-IP analysis was performed as described earlier [4]. MDA-9/Syntenin antibody was procured from Abnova, whereas VEGFR1/SPRRB1 antibodies were procured from Abcam. The quantification of the images was done using Image J software with respect to the appropriate control.

### *In vivo* xenograft analysis

For tumor growth, 1 × 105 transfected cells (FaDu and SCC-15) in 1:1 mixture of PBS and matrigel were injected subcutaneously into athymic, 4-6 week-old male nude mice (Charles River) [6]. All experiments were performed in accordance with the Animal Care and Use Committee guidelines. Each group consisted of 6 mice and each experiment was repeated two times. Mice were examined every day and mice showing signs of morbidity were immediately sacrificed according to University guidelines. After 4 weeks, mice were sacrificed and tumor weights were taken. Tumors were immediately snap frozen as well as processed as FFPE for histological and IHC analysis respectively. Data presented as mean ± S.E. of duplicate experiments.

### Receptor tyrosine kinase (RTK) array

We procured human RTK array blot from R&D Systems (# ARY001B) and perform all the experiments as the manufacturer's protocol. The quantification of the images was done using Image J software with respect to the controls and data represented as mean ± SE of duplicate experiments.

### Angiogenesis array

We procured human RTK array blot from R&D Systems (# ARY007) and perform all the experiments as the manufacturer's protocol. The quantification of the images was done using Image J software with respect to the controls and data represented as mean ± SE of duplicate experiments.

### HUVAC cell tube formation assay

We procured HUVAC cells from Lonza Inc. and performed the tube formation assay using a kit and protocol available from CellBiolabs (# CBA-200). Cells plated on 10cm dishes were treated with conditioned medium obtained from the various transfected groups as per the manufacturer's specification. Tube formation was examined under a brightfield microscope at x 200 magnification.

### Microarray analysis of MDA-9/Syntenin KD FaDu cells

The microarray analyses were done in collaboration with the Department of Pathology, at VCU (C.I.D). Total RNA was extracted from 1×107 FaDu cells stably transfected with empty vector or ShMDA-9 (in triplicates). Cells were re-suspended in 300 μL of Trizol and total RNA was isolated using the MagMAX™-96 for Microarrays Total RNA Isolation Kit (InvitrogenTM Life Technologies, Carlsbad, CA). Microarray analysis was performed in triplicate as described earlier [34-35]. All data were submitted to Gene Expression Omnibus Data Base (#GSE57760).

### FACS analysis

FACS analysis was performed at the VCU core facility.

### Statistical Analysis

Chi-square, Fisher's exact or Student's *t* test tests were used when appropriate. All p-values were two-sided and all confidence intervals were at the 95% level. Computation for all the analysis was performed using the Statistical Analysis System (SAS) (KPS and SB).

## SUPPLEMENTARY MATERIAL FIGURES AND TABLE


