# Supplementary material for: MDA-9/Syntenin regulates differentiation and angiogenesis programs in head and neck squamous cell carcinoma
Source: Oncoscience. 2014 Sep 18;1(11):725–37. doi: 10.18632/oncoscience.99 (PMC4278274; doi:10.18632/oncoscience.99)
Supplement: Supplementary file 1 [file oncoscience-01-0725-s001.pdf]

MDA-9/Syntenin regulates differentiation and angiogenesis programs in head and neck squamous cell carcinoma

Supplementary Material

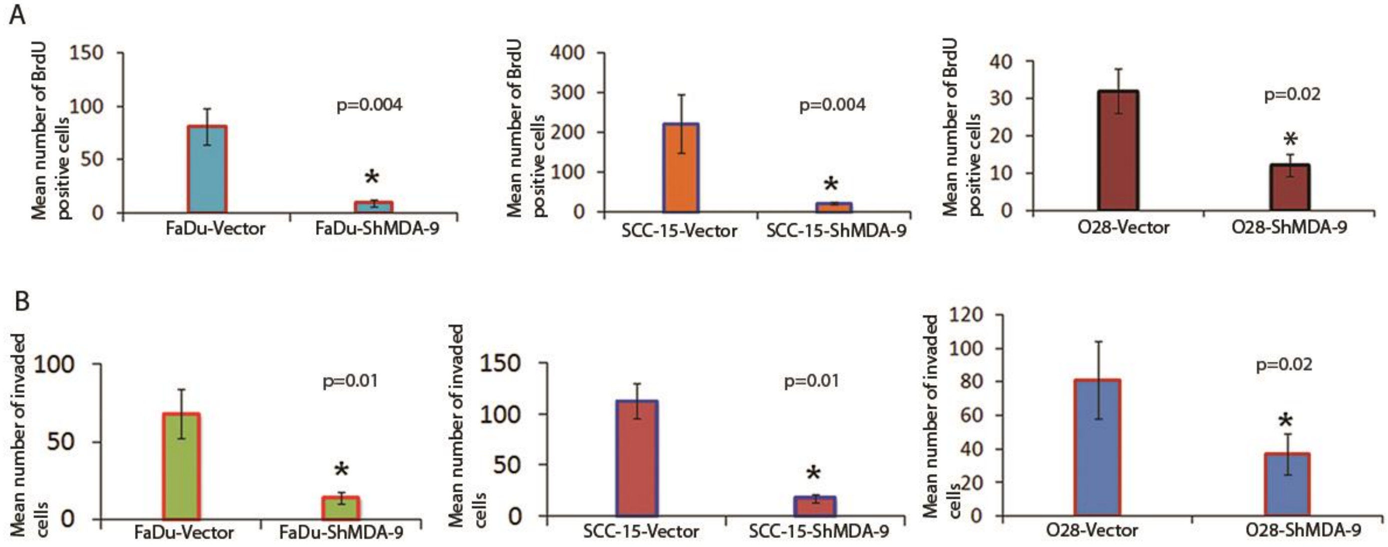

Figure S1: *In vitro* cellular proliferation (*A*, p=0.004-0.02) and invasion (*B*, p=0.01-0.02) was significantly reduced in the MDA-9/Syntenin silenced groups compared to the empty vector treated groups. Each experiment was repeated two times and mean ± SE of duplicate experiments are represented.

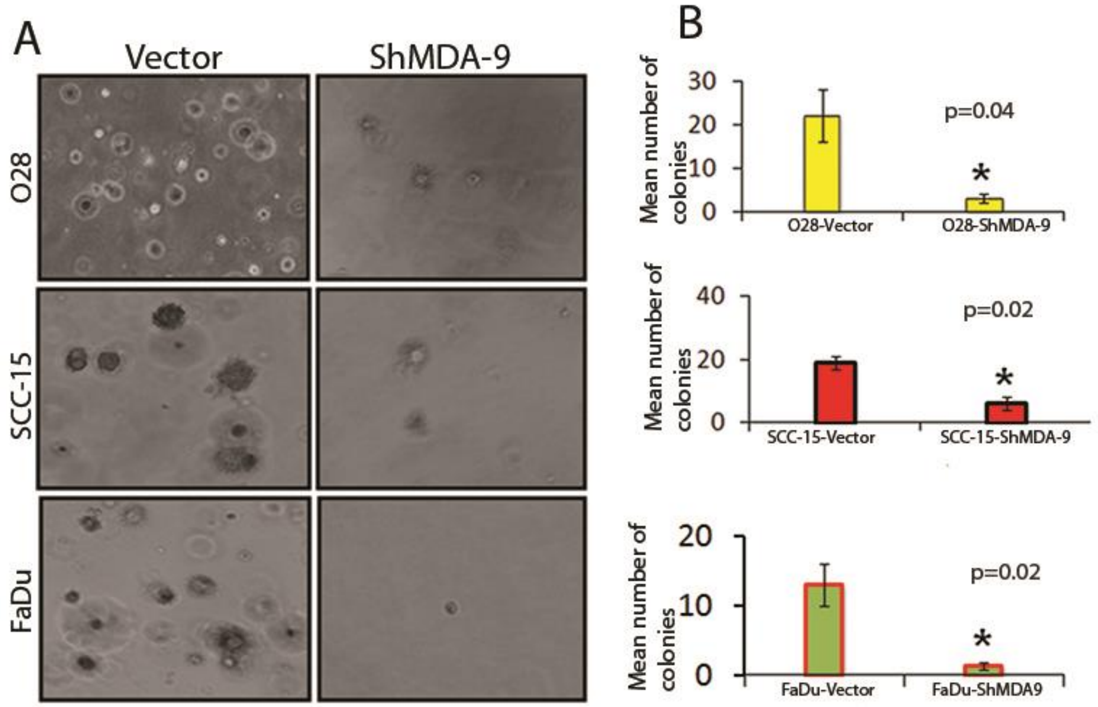

Figure S2: Anchorage independent growth as determined by soft agar assay was significantly reduced (p=0.04-0.02) in the MDA-9/Syntenin silenced HNSCC groups compared to the empty vector treated groups. Each experiment was repeated two times and mean ± SE of duplicate experiments are represented.

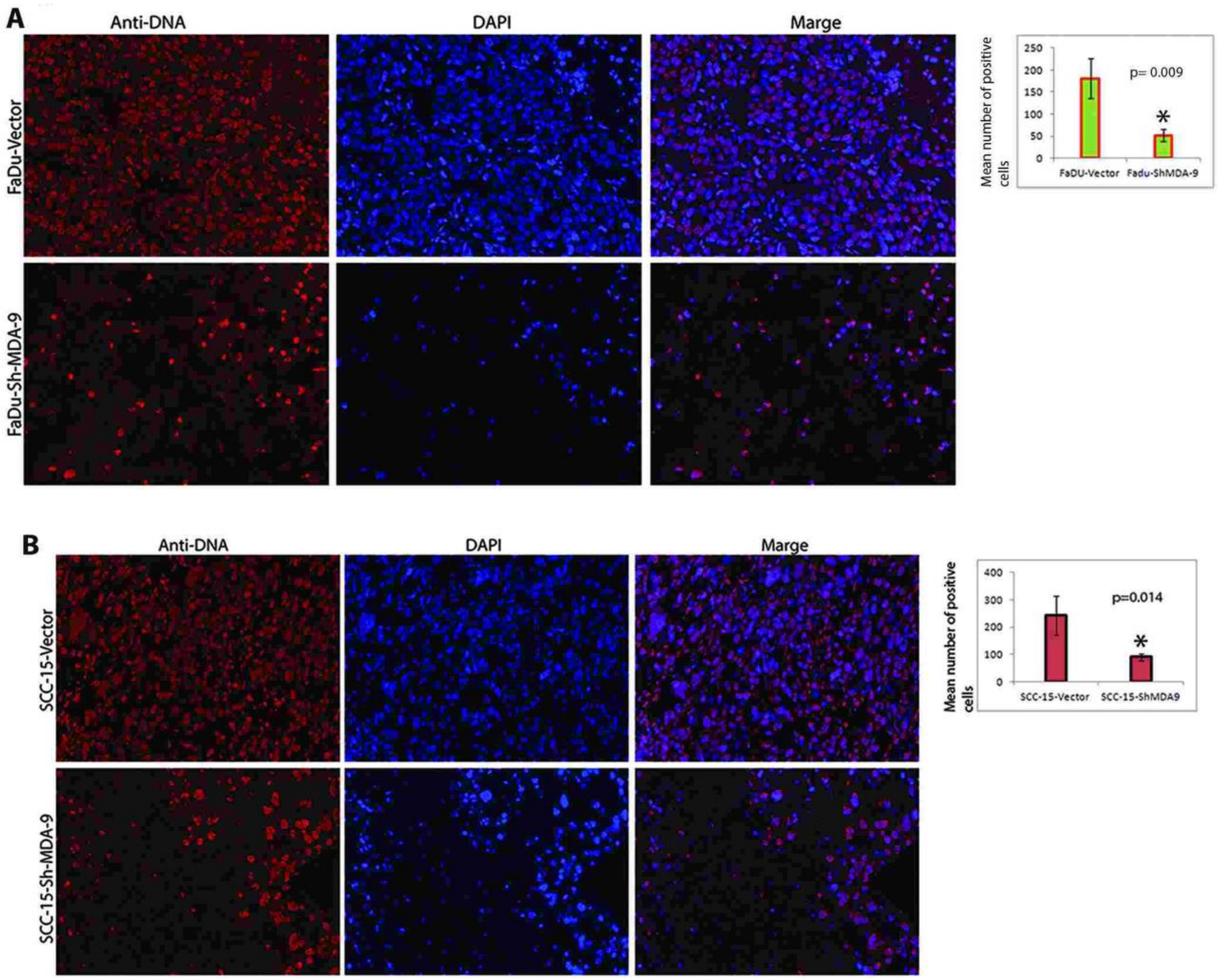

Figure S3: The extent of DNA-synthesis was significantly reduced in the MDA-9/Syntenin depleted FaDu (A, p=0.009) and SCC-15 (B, p=0.014) xenografts compared to the vector treated controls. Tumors from at least 5 mice were examined and data were represented as mean ± SE of counting of 10 randomly selected microscopic fields. Magnification X 200.

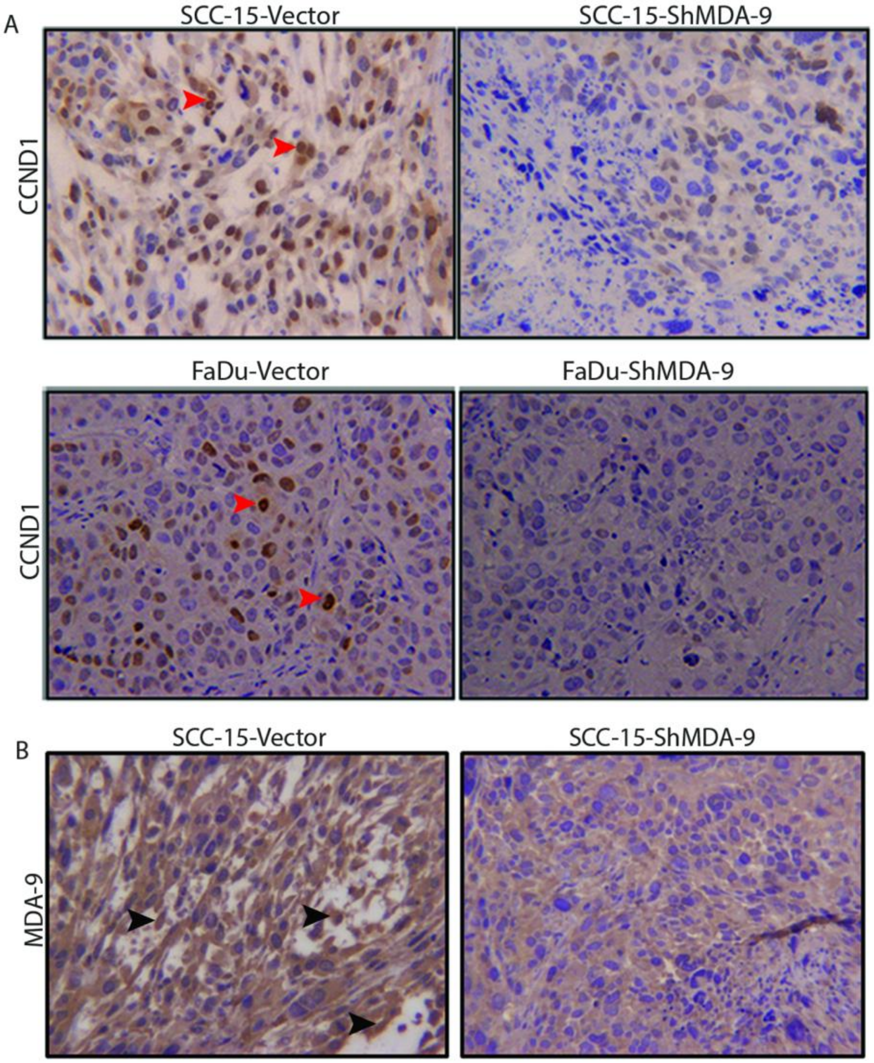

Figure S4: The expression level of CCND1 was significantly reduced (p=0.01-0.03) in the MDA-9/Syntenin depleted FaDu and SCC-15 xenografts compared to the empty vector treated groups (A). The expression level of MDA-9/Syntenin was lower (p=0.01) in the MDA-9/Syntenin depleted SCC-15 xenografts compared to the empty vector treated groups (B). Arrowheads indicate nuclear localization of MDA-9/Syntenin. Magnification X 200.

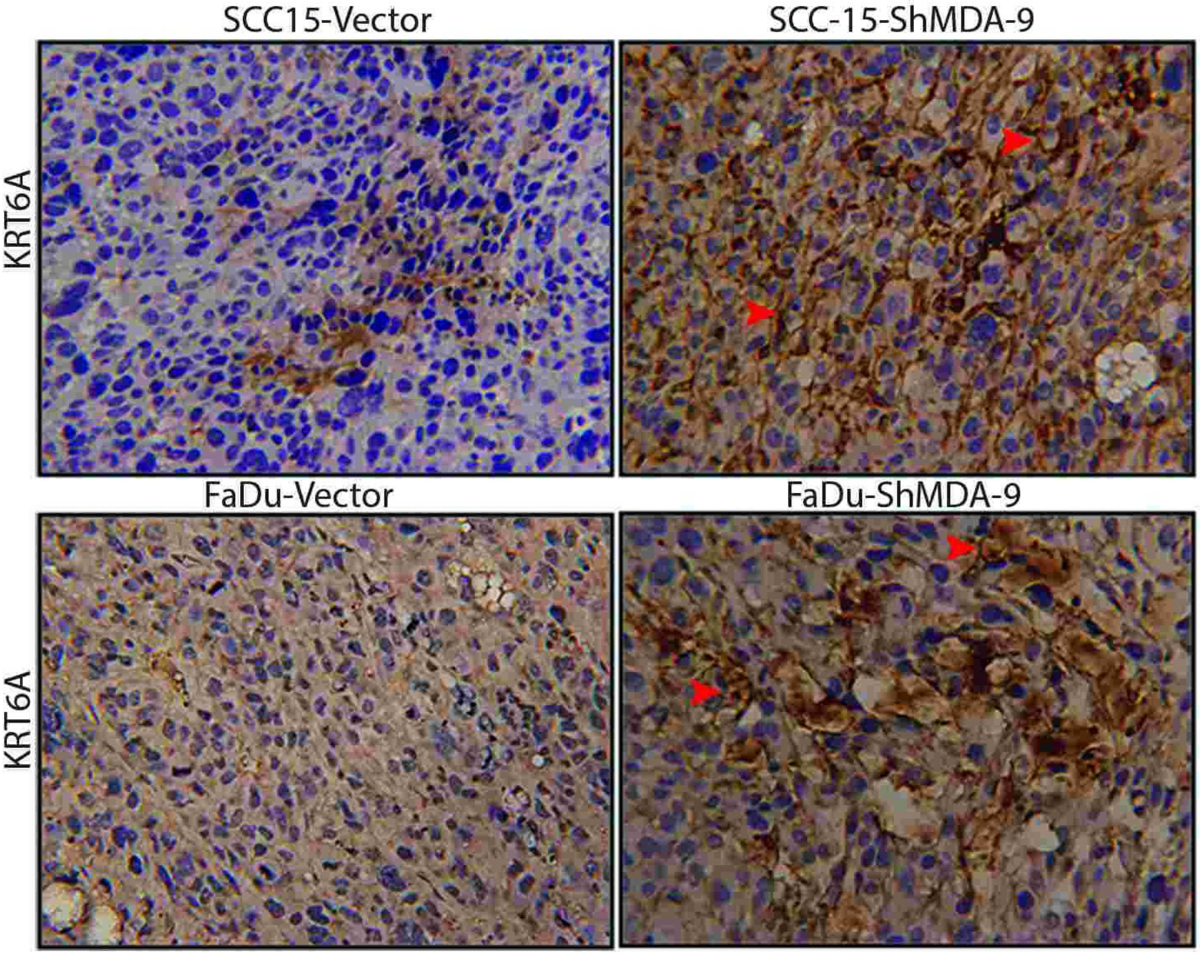

Figure S5: The expression level of KRT6A was significantly increased (p=0.001, arrowheads) in the MDA-9/Syntenin depleted FaDu and SCC-15 xenografts compared to the control vector treated groups. Magnification X 200.

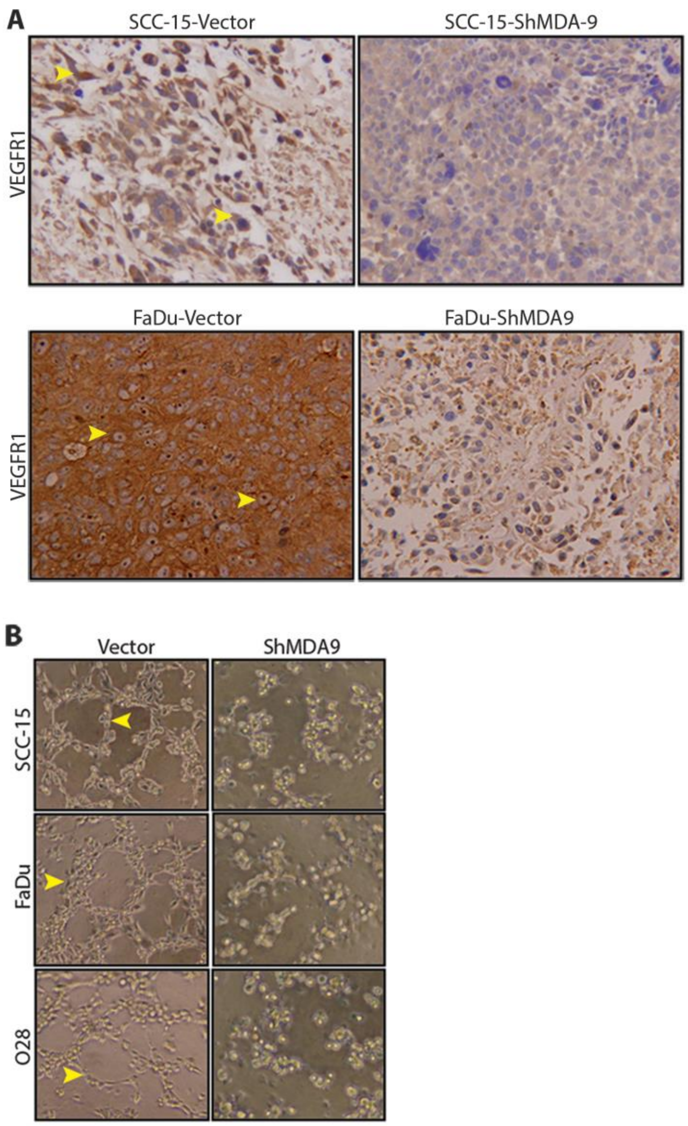

Figure S6: The expression level of VEGFR1 (arrow heads) was significantly reduced (p=0.001-0.003) in the MDA-9/Syntenin depleted FaDu and SCC-15 xenografts compared to the empty vector treated groups (A). (B) HUVAC cells treated with conditioned medium from empty vector treated HNSCC cells resulted in capillary tube formation (left panel, arrow heads). Disrupted capillary tube formation by the HUVAC cells when treated with conditioned medium obtained from MDA-9/Syntenin depleted HNSCC cells (right panel). Magnification x 200.

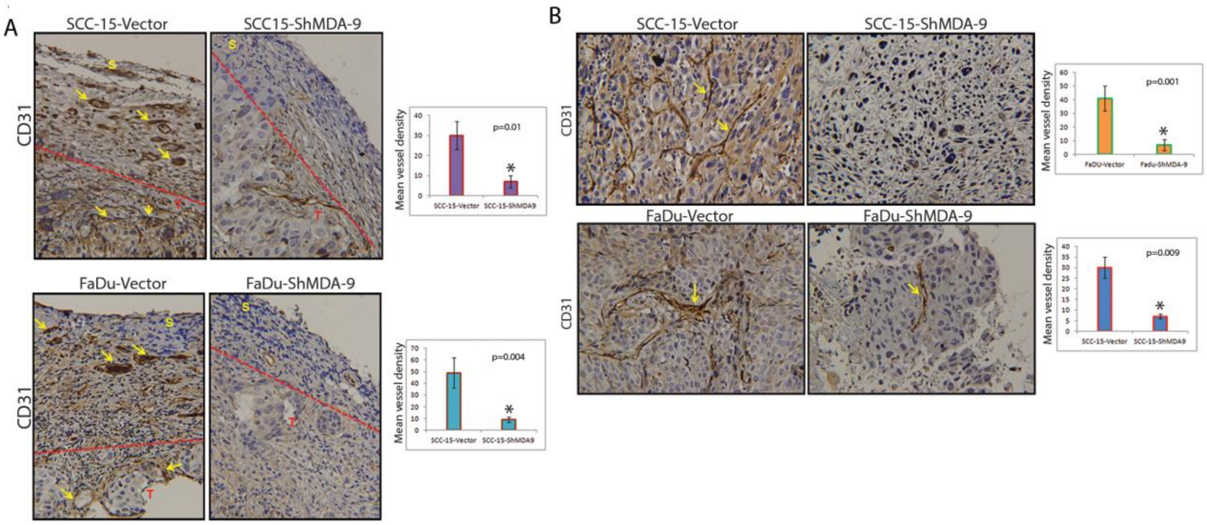

Figure S7: Vasculature of the transfected xenografts. (A) Mean vessel density as determined by CD31 positivity was significantly lower in the tumor surrounding stroma (arrows) of the MDA-9/Syntenin depleted SCC-15 (p=0.01) and FaDu (p=0.004) xenografts compared to the empty vector treated groups. Red dotted lines indicate tumor-stromal interface. S: stroma; T; tumor. Magnification x 200. (B) Mean vessel density as determined by CD31 positivity (arrows) was also significantly lower in the tumor beds of the MDA-9/Syntenin depleted SCC-15 (p=0.001) and FaDu (p=0.009) xenografts compared to the empty vector treated groups. Magnification x 200.

Table S1. Expression level of MDA-9/Syntenin in the primary HNSCC tumors.

Patient ID	Age	Gender[1]	Primary site[2]	TNM[3]	Stage[4]	Grade[5]	LNM[6]	MDA9[7]
HN1	39	M	Tongue	T3N2MX	IV	MDSCC	Y	**SH (+++)**
HN2	35	M	Tongue	T3N1MX	IV	MDSCC	Y	**SH (++++)**
HN3	56	M	Tongue	T4N3MX	IV	PDSCC	Y	**SH (+++)**
HN4	70	F	Oropharynx	T2N2aMx	III	WDSCC	Y	**SH (++++)**
HN5	51	M	Tongue	T2NXMX	II	MDSCC	N	**SH (++)**
HN6	58	M	Tonsil	TXN3MX	III	PDSCC	Y	**SH (+++)**
**HN7**	53	M	Tongue	T4N2cMX	IV	MDSCC	Y	**SH (+++)**
HN8	41	F	Tongue	T2N2bM0	III	MDSCC	Y	**SH (++++)**
HN9	60	M	Larynx	T3N3MX	IV	PDSCC	Y	**SH (++++)**
HN10	77	F	FOM	T3N0M0	III	MDSCC	N	**SH (++)**
HN11	53	M	FOM	T2N0MX	II	MDSCC	N	NSC
HN12	48	M	FOM	T4N2CMX	IV	PDSCC	Y	**SH (+++)**
HN13	57	M	Tongue	NA	NA	MDSCC	NA	**SH (+++)**
HN14	57	M	Larynx	T2N0MX	II	MDSCC	N	**SH (+++)**
HN15	60	M	Tongue	T2N1MX	III	MDSCC	Y	**SH (++++)**
HN16	64	F	FOM	T2N0MX	II	MDSCC	N	NSC
HN17	59	M	Tongue	T3N0MX	III	MDSCC	N	**SH (++)**
HN18	61	M	Larynx	T3N1MX	IV	MDSCC	Y	**SH (+++)**
HN19	54	M	FOM	T3N3M0	IV	MDSCC	Y	**SH (++++)**
HN20	69	M	Tongue	NA	NA	PDSCC	NA	**SH (++)**
HN21	65	M	Larynx	T2N1M0	III	PDSCC	Y	NSC
HN22	52	M	Larynx	T3N2MX	IV	PDSCC	Y	**SH (+++)**
HN23	32	F	Maxilla	T2N0MO	III	MDSCC	N	NSC
HN24	61	M	Larynx	T2N1M0	III	MDSCC	Y	**SH (++++)**
HN25	44	M	Laryngeal pharynx	T2N1M0	III	MDSCC	Y	**SH (+++)**
HN26	61	M	Larynx	T3N0M0	III	MDSCC	Y	**SH (+++)**
HN27	47	M	Larynx	T2N0M0	II	MDSCC	N	NSC
HN28	63	M	Epiglottis	T3N1M0	III	MDSCC	Y	**SH (+++)**
HN29	61	M	Epiglottis	T2N0M0	II	PDSCC	N	**SH (++++)**
HN30	75	M	Maxilla	T4N0M0	IV	PDSCC	N	NSC
HN31	50	M	Maxilla	T2N0M0	II	PDSCC	N	**SH (++++)**
HN32	64	M	Larynx	T2N0M0	II	PDSCC	N	**SH (+++)**
HN33	38	F	Lip	T2N0M0	II	NA	N	NSC
HN34	47	M	Larynx	T2N1M0	III	NA	Y	**SH (+++)**
HN35	51	M	Larynx	T2N1M0	III	MDSCC	Y	**SH (++++)**
HN36	58	M	Maxilla	T2N2M0	IV	NA	Y	NSC
HN37	74	M	Laryngeal pharynx	T4N0M0	IV	PDSCC	N	**SH (+++)**
HN38	48	M	Laryngeal pharynx	T4N0M0	IV	MDSCC	N	**SH (++)**
HN39	65	M	Larynx	T3N2M0	IV	PDSCC	Y	**SH (++)**
HN40	75	M	Larynx	T4N0M0	IV	PDSCC	N	NSC
**HN41**	44	M	Tonsil	T1N1M0	II	MDSCC	Y	**SH (++++)**
HN42	56	F	FOM	T3N0M0	III	PDSCC	N	**SH (++)**
HN43	42	F	Tongue	T3N0M0	III	NA	N	**SH (++)**
HN44	45	M	Tonsil	T2N0M0	II	PDSCC	N	**SH (++)**
HN45	61	F	Palate	T1N0M0	I	WDSCC	N	NSC
HN46	61	M	Larynx	T1N0M0	I	WDSCC	N	**SH (+++)**
HN47	77	M	Larynx	T1N0M0	I	WDSCC	N	NSC
HN48	55	M	Tongue	T1N0M0	I	WDSCC	N	NSC
HN49	67	M	Palate	T2N0M0	II	WDSCC	N	NSC
HN50	67	M	Larynx	T2N0M0	II	WDSCC	N	NSC
HN51	70	M	Maxilla	T2N0M0	II	WDSCC	N	NSC
HN52	40	M	larynx	T2N1M1	IV	MDSCC	Y	**SH (+++)**

HN53	55	M	Epiglottis	T4N0M0	IV	MDSCC	N	**SH (+++)**
HN54	57	M	Pharynx	T4N0M0	IV	MDSCC	N	**SH (++)**
HN55	49	M	Gingiva	T4N0M0	IV	WDSCC	N	NSC
HN56	50	M	Tongue	T3N0M0	III	MDSCC	N	**SH (+++)**
HN57	72	M	Larynx	T2N0M0	II	MDSCC	N	**SH (++)**
HN58	50	M	Tongue	T2N0M0	II	NA	N	NSC
HN59	49	M	Larynx	T4N1M0	IV	MDSCC	Y	**SH (++)**
HN60	50	M	Larynx	T2N1M0	III	MDSCC	Y	**SH (++)**
Hn61	90	M	FOM	T2N0M0	II	MDSCC	N	NSC
HN62	67	M	Larynx	T3N1M0	III	MDSCC	Y	**SH (++++)**
HN63	56	M	Epiglottis	T4N0M0	IV	MDSCC	N	**SH (++++)**
HN64	66	M	Laryngeal pharynx	T3N2M0	IV	MDSCC	Y	**SH (++)**
HN65	58	M	Larynx	T3N1M1	IV	MDSCC	Y	**SH (+++)**
HN66	60	M	Tongue	T2N0M0	II	PDSCC	N	NSC
HN67	67	M	Maxilla	T2N0M0	II	PDSCC	N	NSC
HN68	56	F	Nasopharynx	T2N0M0	II	PDSCC	N	**SH (++)**
HN69	48	M	Submaxilla	T2N0M0	II	WDSCC	N	NSC
HN70	56	M	Laryngeal pharynx	T2N0M0	II	MDSCC	N	NSC
HN71	43	M	Submaxilla	T2N0M0	II	MDSCC	N	**SH (++)**
HN72	70	M	Submaxilla	T2N0M0	II	MDSCC	N	NSC
HN73	61	F	Submaxilla	T2N0M0	II	MDSCC	N	**SH (++)**
HN74	56	F	FOM	T2N0M0	II	MDSCC	N	NSC
HN75	55	M	Larynx	T3N1M0	III	MDSCC	Y	**SH (+++)**
HN76	50	M	Larynx	T3N1M0	III	MDSCC	Y	**SH (+++)**
HN77	49	M	Larynx	T2N1M0	III	MDSCC	Y	NSC
HN78	71	M	Epiglottis	T3N1M0	III	MDSCC	Y	**SH (+++)**
HN79	50	F	Laryngeal pharynx	T2N1M0	III	MDSCC	Y	NSC
HN80	58	M	Maxilla	T3N0M0	III	MDSCC	N	NSC
HN81	71	M	Laryngeal pharynx	T2N1M0	III	MDSCC	Y	**SH (+++)**

[1]M: Male; F: Female; [2]FOM: Floor of mouth; [3]T: primary tumor, N; regional lymph node involvement, M: distant metastasis; [4]Clinical staging based on TNM status, NA: not available; [5]WDSCC: Well differentiated squamous cell carcinoma, MDSCC: Moderately differentiated squamous cell carcinoma, PDSCC: Poorly differentiated squamous cell carcinoma, NA: not available; [6]LNM: lymph node metastasis, Y: Yes, N: No; [7]Expression level of MDA-9 determined by immunohistochemistry per pathologic guidance and scoring, SH: significantly higher expression of MDA-9 compared to matched tumor-free normal control. NSC: No significant change in the expression of MDA-9 compared to control. In all cases, ≥70% tumor tissues in randomly selected microscopic fields, overexpressed MDA-9 at variable levels, which were represented as fold increase and denoted by + sign (value range 129-287) compared to the corresponding normal (10-15% positive, value range 0-50, represented by single + sign). Mean intensity values were then compared between matched normal and tumor by student *t* test to infer significance.
